# Supplementary material for: FastST: an efficient tool for inferring decomposition and directionality of microbial communities
Source: PeerJ. 2025 Oct 27;13:e20161. doi: 10.7717/peerj.20161 (PMC12574587; doi:10.7717/peerj.20161)
Supplement: Supplemental Information 1 [file peerj-13-20161-s001.pdf]

Supplementary Material S1.
Average Jensen-Shannon divergence and Pearson correlation for estimating the proportion of sources in fully simulated microbiome data across MST methods.

Average Jensen–Shannon divergence for estimating proportions of sources						
Number of known sources	Number of major sources	FastST	FEAST	SourceTracker2	STENSL	SourceID-NMF
2	2	0.1669	0.0921	0.2462	0.1530	0.1324
5	2	0.0784	0.1473	0.3143	0.2042	0.3130
5	5	0.1768	0.0493	0.1692	0.1549	0.0567
10	2	0.0611	0.1523	0.3463	0.1570	0.3723
10	5	0.0715	0.1377	0.2957	0.2001	0.2356
50	2	0.1224	0.1559	0.5719	0.1944	0.2543
50	5	0.1262	0.1556	0.5775	0.1904	0.2116
100	2	0.1794	0.1563	0.6684	0.8116	0.2451
100	5	0.1852	0.1622	0.6513	0.5865	0.2228

Average Pearson correlation for estimating proportions of sources						
Number of known sources	Number of major sources	FastST	FEAST	SourceTracker2	STENSL	SourceID-NMF
2	2	0.9890	0.9798	0.1338	0.8663	0.9772
5	2	0.9973	0.9826	0.8029	0.9377	0.7774
5	5	0.9295	0.9375	0.0439	0.3132	0.9831
10	2	0.9994	0.9902	0.9534	0.9828	0.7102
10	5	0.9946	0.9640	0.7398	0.8142	0.8108
50	2	0.9987	0.9985	0.9541	0.9966	0.9783
50	5	0.9987	0.9955	0.7781	0.9900	0.9877
100	2	0.9995	0.9995	0.8115	-0.0254	0.9954
100	5	0.9983	0.9981	0.5183	0.3664	0.9947
