# Supplementary material for: FastST: an efficient tool for inferring decomposition and directionality of microbial communities
Source: PeerJ. 2025 Oct 27;13:e20161. doi: 10.7717/peerj.20161 (PMC12574587; doi:10.7717/peerj.20161)
Supplement: Supplemental Information 2 [file peerj-13-20161-s002.pdf]

Supplementary Material S2.

Average Jensen-Shannon divergence and Pearson correlation for estimating the proportion of sources in semi-synthetic microbiome data across MST methods.

Average Jensen–Shannon divergence for estimating proportions of sources						
Number of known sources	Number of major sources	FastST	FEAST	SourceTracker2	STENSL	SourceID-NMF
2	2	0.1915	0.0956	0.0840	0.5293	0.3438
5	2	0.1361	0.2646	0.0559	0.6769	0.4705
5	5	0.2017	0.1436	0.0830	0.6873	0.3844
10	2	0.1862	0.2854	0.0869	0.6987	0.4980
10	5	0.1674	0.2905	0.1754	0.7847	0.4989
50	2	0.4290	0.3353	0.2072	0.7605	0.2296
50	5	0.4239	0.3113	0.2569	0.8138	0.2674

Average Pearson correlation for estimating proportions of sources						
Number of known sources	Number of major sources	FastST	FEAST	SourceTracker2	STENSL	SourceID-NMF
2	2	0.9863	0.9636	0.9804	-0.4990	-0.4144
5	2	0.9910	0.8614	0.9986	-0.1350	0.2616
5	5	0.8499	0.2548	0.7666	-0.4756	-0.3401
10	2	0.9887	0.9186	0.9988	0.0096	0.3971
10	5	0.9435	0.5697	0.9426	-0.2512	0.0464
50	2	0.9265	0.9760	0.9968	0.0395	0.9806
50	5	0.8100	0.9503	0.9862	-0.0349	0.9462
