# Supplementary material for: FastST: an efficient tool for inferring decomposition and directionality of microbial communities
Source: PeerJ. 2025 Oct 27;13:e20161. doi: 10.7717/peerj.20161 (PMC12574587; doi:10.7717/peerj.20161)

**Tutorial for the FastST software**

1. **Introduction**

FastST is a microbial source tracking tool which makes fast inference for the relative contributions of source environments to sink microbiomes.

1. **Preparation**

Before running the script, you need to

1. make sure R packages "npreg" and "gtools" have been installed. If not, please run the following lines in R:

install.packages("npreg")

install.packages("gtools")

1. make sure "FastST" is the current working directory. If not, please modify the following line using your home directory and run it in R:

setwd("C:/Users/username/Documents/Work/FastST")

1. make sure environment variables are correctly set, e.g., for windows “C:\Program Files\R\R-4.5.1\bin” is included.
2. If you are running our tools in Linux or Mac, please change the permission for the standalone files running “chmod +x standalone_file” command, for example :
   chmod +x ./Code/FastST
   chmod +x ./Code/Demo_GenData
   chmod +x ./Code/Demo_EstProp
3. Code description

All code, including R scripts and standalone commands, are listed in the following table:

| For demonstration | For simulation | For real data analysis |
| --- | --- | --- |
| Demo.R  Demo_GenData  Demo_EstProp | Simu_Scen1a.R  Simu_Scen1b.R  Simu_Scen2.R | Real.R  FastSt |

--Demo.R: R script for demonstrating a simple example of generating sink/source data and estimating proportions of source contribution.

--Demo_GenData: standalone command for demonstrating the data generation procedure.

--Demo_EstProp: standalone command for demonstrating the proportion estimation procedure.

--Simu_Scen1a.R: R script for Scenario 1a (proportion estimation) with fully simulated microbiome data.

--Simu_Scen1b.R: R script for Scenario 1b (directionality inference) with fully simulated microbiome data.

--Simu_Scen2.R: R script for Scenario 2 with semi-synthetic data generated from the Knights et al. dataset.

study.

--Real.R: R script for real data analysis based on the Knights et al. dataset.

--FastSt: standalone command for analyzing user specified data.

Note: Users may simply run “**./Code/FastST userdata.txt**” to analyze their own microbiome data. The file “userdata.txt” contains an N by K matrix where the 1^st^ column is the observed sink counts on N taxa, and the rest columns are the counts of the observed K sources.

After running FastST, a “Result” directory will be created in the current working directory, containing the output file “**./Result /results.txt**”. In this file, the first column represents the estimated proportions computed by FastST, and the second column shows the standard errors. The first row corresponds to the unknown source.

1. **Demonstration.**

The demo code contains two parts: generating sink data and estimating source proportions. It can be executed in two different modes:

1.
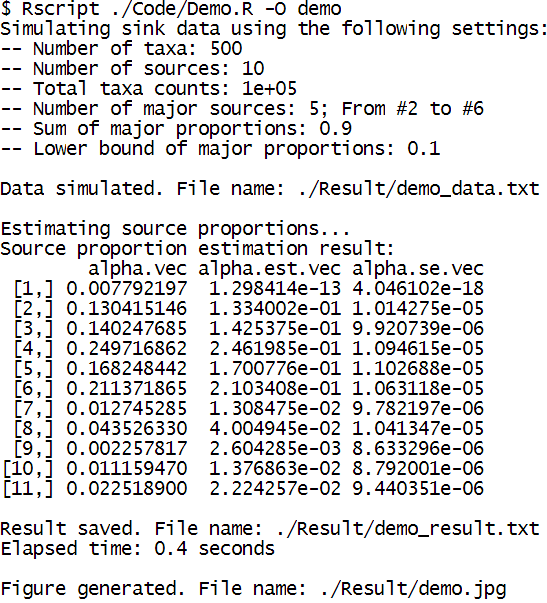
as one R script, please run "./Code/Demo_FastST.R". For example, in RStudio terminal pane (first, you will need to set correct environment variables in the Shell for the Rscript command),

Below is the generated figure “demo.jpg”

| 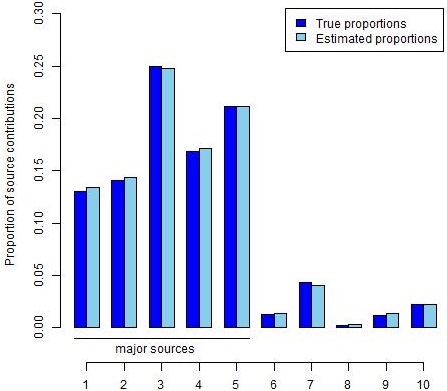 |
| --- |

For more help, please see

| 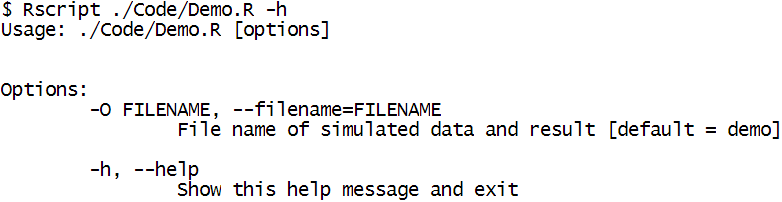 |
| --- |

1. as two standalone commands which allow the specification of input arguments, please run the following commands in RStudio terminal pane:

| 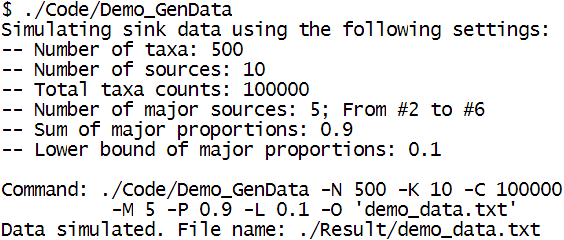 |
| --- |
| After running Demo_GenData, “./Result/demo_data.txt”,   will be created, where the first column represents the   sink and the rest column represent the sources. |
| 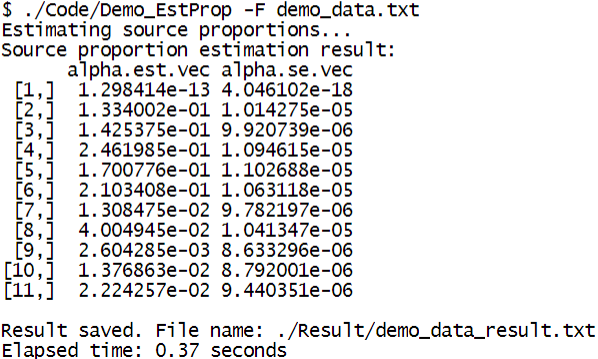 |

1. Simulation.

The simulation study contains three parts:

1. Scenario 1a (proportion estimation) with fully simulated microbiome data, please run the following commands in RStudio terminal pane:


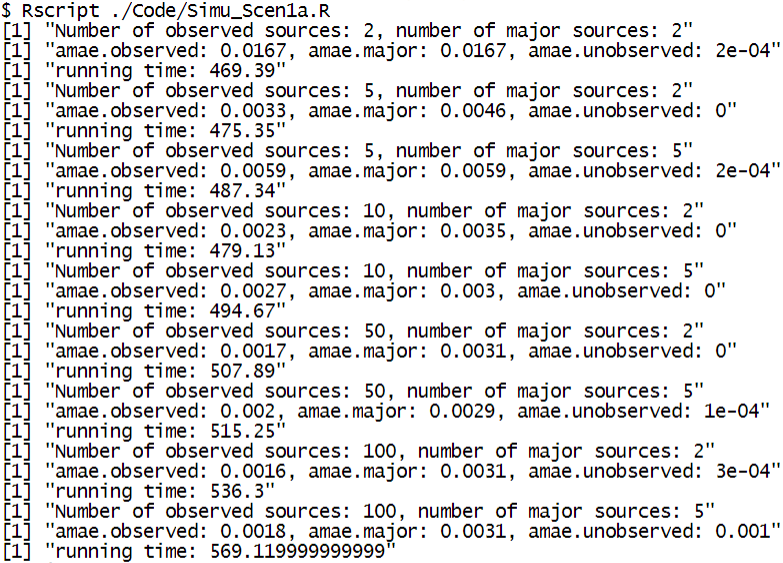


1. Scenario 1b (directionality inference) with fully simulated microbiome data, please run the following commands in RStudio terminal pane:


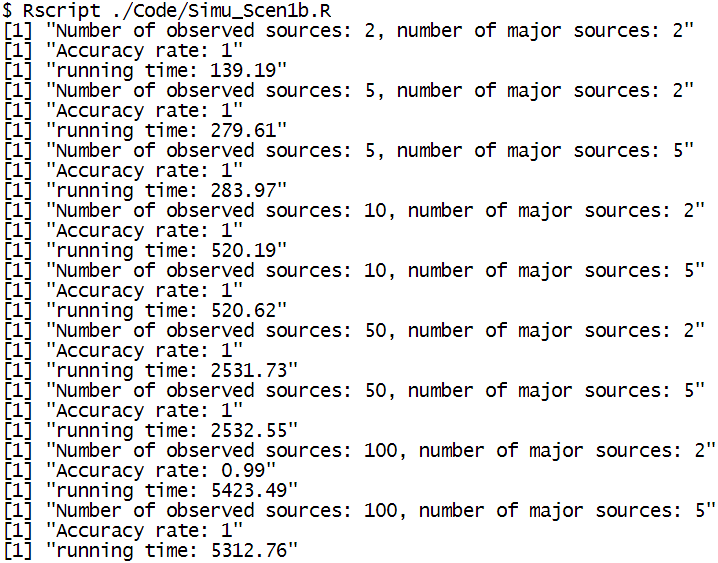


1. Scenario 2 with semi-synthetic data generated from the Knights et al. dataset, please run the following commands in RStudio terminal pane:


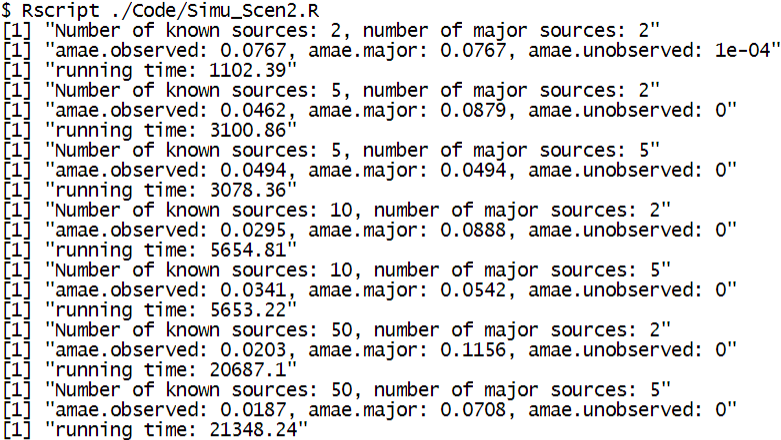


1. Real data analysis using the Knights et al. dataset. Please run the following commands in RStudio terminal pane:


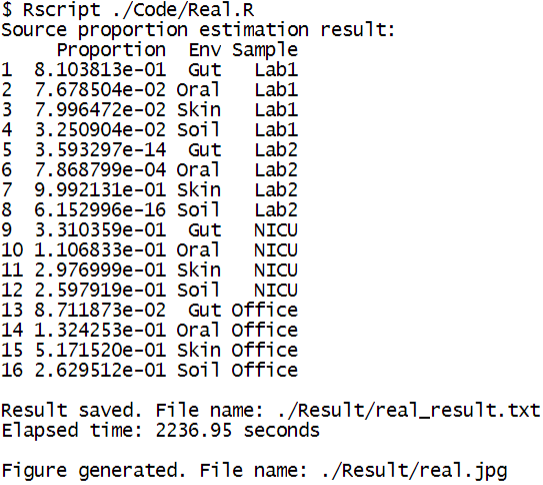

Supplement: Supplemental Information 3 [file peerj-13-20161-s003.docx]
